# Merging the Psychophysical Function With Response Times for Auditory Detection of One vs. Two Tones

**DOI:** 10.3389/fpsyg.2022.910740

**Published:** 2022-09-08

**Authors:** Jennifer J. Lentz, James T. Townsend

**Affiliations:** ^1^Department of Speech, Program in Cognitive Sciences, Language and Hearing Sciences, Indiana University, Bloomington, IN, United States; ^2^Department of Psychological and Brain Sciences, Program in Cognitive Sciences, Indiana University, Bloomington, IN, United States

**Keywords:** loudness, Systems Factorial Technology (SFT), reaction times (RTs), coactivation, architecture, workload capacity, tone detection, power law

## Abstract

The purpose of this study is to take preliminary steps to unify psychoacoustic techniques with reaction-time methodologies to address the perceptual mechanisms responsible for the detection of one vs. multiple sounds. We measured auditory redundancy gains for auditory detection of pure tones widely spaced in frequency using the tools of Systems Factorial Technology to evince the system architecture and workload capacity in two different scenarios (SOFT and LOUD). We adopted an experimental design in which the presence or absence of a target at each of two frequencies was combined factorially with two stimulus levels. Replicating previous work, results did not allow an assessment of system architecture due to a failure to observe factor influence at the level of distribution ordering for dual-target stimuli for both SOFT and LOUD scenarios. All subjects demonstrated very modest redundancy gains for the dual-target compared to the single-target stimuli, and results were similar for both LOUD and SOFT. We propose that these results can be predicted by a mental architecture that falls into the class of integrated subadditive parallel systems, using a well-supported assumption that reaction time is driven by loudness. We demonstrate that modeled loudness of the experimental sounds (which ranged between about 0.2 and 14 sones) is highly correlated with mean reaction time (*r* = −0.87), and we provide a proof-of-concept model based on Steven’s Power law that predicts both a failure of distributional ordering for dual-target stimuli and very modest redundancy gains.

## Introduction

An historical interest in the field of auditory perception is the characterization of the mechanisms responsible for the monaural detection of complex sounds. The psychoacoustic approach typically measures detection thresholds or percent correct detections (i.e., accuracy) whereas a second approach measures the reaction time (RT) to a stimulus. Both approaches have been applied to monaural auditory detection of one vs. two (or many) tones. Psychoacoustic experiments evaluate mechanisms at near-threshold levels, whereas RT measures are generally (although not exclusively) used under conditions of high accuracy. So far, most of the RT studies have focused on mean RTs, with the exception of certain work exploring the phenomena associated with redundant signals (e.g., Schröter et al., 2007, 2009; Fiedler et al., 2011; Lentz et al., 2016, 2017).

It is our position that with certain exceptions, RTs and accuracy methodologies each have advantages regarding important questions involving human information processing. For instance, to take a simple but critical example, consider the detection of two tones vs. a single tone. In psychoacoustic studies, it seems to be presumed that probabilistically independent detection when two tones are present is tantamount to parallel processing. This is a sufficient cause, but independent serial processing is not at all ruled out, based on response frequencies (e.g., percent correct or accuracy) alone. In many cases, RTs provide stronger means of assessing mental architecture (e.g., serial vs. parallel processing; Townsend and Ashby, 1983). Likewise, response frequency measures allow a rigorous assessment regarding independence of features, dimensions, etc. This realization has led to two distinct theory-driven methodologies: Systems Factorial Technology (SFT; Townsend and Nozawa, 1995; Little et al., 2017) in the case of RTs and General Recognition theory in the case of response frequencies (Ashby and Townsend, 1986; Ashby, 1992; Townsend et al., 2012).

Regarding the mechanisms underlying detection or discrimination for one vs. multiple tones, detection being the issue facing us in this study, psychoacoustic approaches generally support an integration (more on integration shortly) model in which the representations of the tones (i.e., the observations) are combined into a single decision variable (e.g., Green, 1958; Green et al., 1959), such as that described by Signal Detection Theory (SDT; Tanner and Swets, 1954; Green and Swets, 1966). Such work applies predominantly to near-threshold sound levels, and may not generalize to the detection of sounds at supra-threshold levels. The literature on high-accuracy RTs (which are measured at supra-threshold levels) regarding this issue is more recent and relatively sparse (Schröter et al., 2007, 2009; Fiedler et al., 2011; Lentz et al., 2016, 2017) and that which treats both RTs and accuracy in parathreshold domains is more exiguous still. This is surprising given that there is now a healthy and still growing body of knowledge on stochastic models of information processing and decision-making.

To date, it remains unknown to what degree RT and SDT models will fruitfully inform each other regarding detection of one vs. many tones in acoustics, and it may not be straightforward to generalize the SDT structure obtained from accuracy-based experiments to the RT domain, or vice versa. For example, SDT has been successful with an assumption that two random variables are added with equal weights of magnitude 1 followed by comparison with a decision criterion (c.f. Green, 1958; Buus et al., 1986; Van den Brink and Houtgast, 1990a,b; Hicks and Buus, 2000). It might appear that a natural extension of this concept to dynamic and high accuracy experiments would be to assume that two independent random signals are summed (one from each of two tones) and this combined signal is then continuously compared with a decisional criterion. However, it turns out that such a model predicts a response time that is much faster than ordinary parallel processing with independent channels (a result referred to as *super* capacity by Townsend and Nozawa, 1995, to be dealt with in more detail below). For example, beginning with the seminal work of Miller (1978) it has been found many times that, for example, when a sound plus a visual signal are presented together rather than separately, RTs can be speeded up so much that a high upper bound (now known as the race inequality or the *Miller bound*) is violated. In point of fact, Townsend and Nozawa (1995) showed that such violations can only occur at a very high level of super capacity. We will discuss some previous evidence as well as new data that call this strong prediction into question.^1^

In fact, we note that repeated RT studies have not provided evidence in support of super capacity for detection of two tones, including work conducted in collaboration with Miller (Schröter et al., 2007, 2009; Fiedler et al., 2011). In a similar vein, Lentz et al. (2016, 2017) adopted SFT, an approach specifically designed to assess architecture (parallel vs. serial) and workload capacity (how efficiency changes as workload increases). However, they encountered challenges while applying SFT to the detection of one vs. two acoustic stimuli and were unable to assess the resident architecture. They, like Schröter et al. (2007, 2009), also did not affirm the super capacity prediction. In fact, performance fell into the range of *limited* capacity, and the data were sufficiently consistent across observers that we cannot simply relegate these findings to the dustbin of “failed hypotheses.” Clearly, an immediate and apparently parsimonious extension of SDT concepts to the RT domain is hazardous at best, and it can be appreciated that we cannot just pop concepts based on accuracy that are well-accepted in the parathreshold milieu over into that appropriate for high accuracy RT data. In fact, Schröter et al. (2007, 2009) argued that an architecture capable of producing such a result was one in which the random variables based on the observations were integrated (of limited rather than super capacity) into a single decision variable. More detail on these kinds of data and analyses will follow later in the paper.

There is one vein of perceptual research that was not mentioned above but might bear on the emerging picture of how sound intensity is related to RT performance, especially in high accuracy situations. We refer to the classical psychophysical scaling literature. In this investigation, we begin to explore potential connections between the RT literature, both descriptive and theoretical and the psychophysical scaling approaches associated with pioneers like Gustav Fechner, and in particular, S. S. Stevens. It is anticipated that one major benefit is explanation of our (so far) failure to effectively utilize the full potential of SFT when applied to auditory detection tasks.

### Experimental Approaches

Although accuracy-based studies (percent correct or threshold measurements) were the first to make measurements for monaural detection of one vs. two tones (e.g., Schafer and Gales, 1949; Green, 1958; Green et al., 1959), detection was commonly measured in the presence of noise and the methodologies typically adopted forced-choice techniques. Whereas these detection experiments only evaluated performance and perceptual mechanisms near threshold, reaction time experiments measured detection of one vs. two tones at supra-threshold levels. The first application of RT to auditory detection that we are aware of was conducted by Schröter et al. (2007), who measured detection in quiet (not in noise, as in the aforenoted psychophysical studies) of a 300-ms, 60 dB SPL pure tone presented to the left ear, the right ear, or both ears. Whether the two tones presented to the different ears had identical or different frequencies, there was a small *redundant-signal benefit.* RTs were faster for detecting two tones vs. one tone, but the decrease in RT was less than would be expected under the statistical advantage provided by two observations over one in the presence of independence. This purely statistical advantage will be interpreted as *unlimited capacity* below. Schröter et al. (2007) argued that fusion of the two tones into an integrated percept was sufficient to abolish a strong redundant-signal benefit, an interpretation further supported by Schröter et al. (2009) and Fiedler et al. (2011).

In an extension of this work, Lentz et al. (2016, 2017) presented two tones to the same ear or different ear, respectively, but applied the tools of System Factorial Technology (Townsend and Nozawa, 1995) to measure the decision architecture underlying the detection process. They, like Schröter et al. (2007, 2009), found a small redundant-signal benefit, but they were unable to measure the architecture due to the experimental results not supporting some key assumptions needed to determine the architecture. These issues and their implications are discussed in more detail below.

The tools of System Factorial Technology are quite powerful in their ability to determine the architecture of processing of one vs. many observations. They also use somewhat different terminology than that of traditional psychophysics. As a result, we describe the tools and then will return to the findings of the RT studies through the lens of System Factorial Technology.

### Systems Factorial Technology^2^

Much as SDT has been used to predict detection of multiple vs. single tones, SFT also focuses on multiple signals vs. a single signal (e.g., two tones vs. one tone) but uses reaction time (RT) as the dependent variable. The strong tools within this approach can assist an investigator in unveiling the dynamics of the underlying perceptual system and can provide strong tests of the architecture underlying the detection of one vs. two signals.

#### Architecture

**FIGURE 1:**
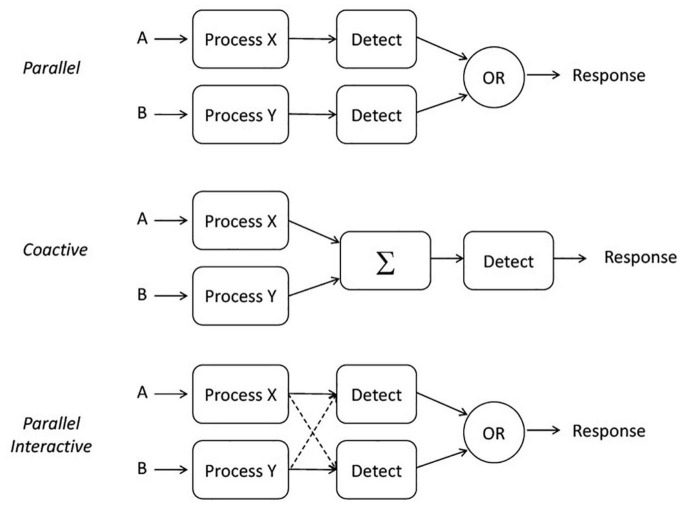
Illustration of three potential architectures: parallel, coactive, and parallel interactive.

A primary component of SFT is to address the form, or the architecture, used by a system. We define three parallel architectures here (illustrated in Figure 1), as these are the most likely benchmarks for comparison with the aforementioned psychoacoustic experiments. *Parallel processing* means processing all the stimuli simultaneously, although each process may finish at different times (e.g., Townsend and Ashby, 1983; Townsend and Eidels, 2011). The model described here includes an OR gate and therefore depicts a parallel *first-terminating* model that stops when the fastest channel completes processing. First termination is a special case of *self-termination* and leads to the minimum-time statistic (e.g., Townsend and Ashby, 1983). *Parallel coactive processing* (or simply *coactive* for short) refers to a system in which the channels are summed together (in unweighted fashion) before a decision is made (Townsend and Nozawa, 1995; Houpt and Townsend, 2011). Lastly, *parallel interactive processing* refers to a system in which each process may have either a facilitatory or inhibitory influence on the other.

Sternberg (1969) invented the notion of “*selective influence*,” also referred to as factorial influence, which stipulated that each experimental factor affects one and only one psychological subprocess at the level of mean RT. Schweickert (1978) was the first to begin generalizing the use of selective influence to other architectures. Most of the theorems were at the deterministic level but a subsequent study provided some bounds in stochastic models of complex networks (so called PERT networks; Schweickert, 1978). Townsend and colleagues later showed that if selective influence acted at the level of distributional ordering, many different architectures, including parallel and serial ones, could be discriminated at the level of mean response times (Townsend and Ashby, 1983; Townsend, 1984, 1990; Schweickert and Townsend, 1989; Townsend and Schweickert, 1989). Selective influence at the level of the RT distributions turned out to provide even stronger inferences, in fact predicting entire functions of RT for the various architectures and stopping rules (Townsend and Nozawa, 1995; Townsend and Wenger, 2004). Even later studies applied these notions to some of the original complex networks first investigated by Schweickert (e.g., Dzhafarov et al., 2004).

Note too that this framework allows for interactions among channels, as illustrated in the bottom panel of Figure 1. In a parallel system, speeding up *A* could either speed up or slow down *B* because they are being processed simultaneously; ongoing inhibition or facilitation (or both) can take place during a single trial and while processing is ongoing. Notably, strong interactions between channels could manifest themselves in a loss of measurable selective influence. Moreover, limited resources could also slow down the channel’s processing speed even though their operations remain stochastically independent. Less likely (but perhaps possible according to Kahneman, 1973), is the possibility that some reservoir partly or completely compensates for an increased workload by increasing the channel efficiencies but again, maintains stochastic independence.

The above matters receive more attention and articulation in the discussions of decisional stopping rule and capacity below.

#### Stopping or Decision Rule: When Does Processing Cease?

Predictions cannot be made about processing times without a rule for when processing stops. As noted above, in any task where a subset of the items (e.g., tones) is sufficient to stop without error, and the system processor is capable of stopping as soon as the first item is processed, the processor is said to be capable of *first-termination*. This case is often called an OR design (as illustrated in Figure 1) since completion of any of a set of presented items is sufficient to stop processing and ensure a correct response (e.g., Egeth, 1966; Townsend and Nozawa, 1995; Townsend and Colonius, 1997).

In the parallel examples of Figure 1, either *A* or *B* could stop processing—both inputs need not be received by the decision element for a decision to be made. This type of architecture together with the OR rule, is commonly called a *race model*, as detection is determined by the first input to reach the decision element (see, e.g., Townsend and Ashby, 1983; Smith and Van Zandt, 2000; Townsend and Liu, 2020).

Using these techniques, we can evaluate the architecture as long as we have evidence for selective influence. A common method to evaluate architecture uses a double-factorial design, in which the two experimental variables (*A* and *B*) are presented at two different levels (1 and 2). The levels should be selected as to manipulate RTs (e.g., level 2 might be associated with a faster RT than level 1; that is level 2 might be a High intensity and level 1 might be a Low intensity). To evaluate architecture, the two variables are presented together at each combination of levels (*A_*H*_B_*H*_, A_*H*_B_*L*_, A_*L*_B_*H*_, A_*L*_B_*L*_*). For example, $\bar{R\,T}$_*HH*_ is defined as the mean RT for when *A* is presented at the High level and *B* is also presented at the High level.

We then define the *mean interaction contrast* as $M\,I\,C=\left({\bar{R\,T}}_{L\,L}-{\bar{R\,T}}_{H\,L}\right)-\left({\bar{R\,T}}_{L\,H}-{\bar{R\,T}}_{H\,H}\right).$ Selective influence at the level of the means, as prescribed by Sternberg requires that ${\bar{R\,T}}_{H\,H}<{\bar{R\,T}}_{H\,L}\approx {\bar{R\,T}}_{L\,H}<{\bar{R\,T}}_{L\,L}$, where we use the ≈ to indicate “approximately equal.” The middle two numbers need not be ordered though it is not prohibited. In any event, the MIC statistic does not distinguish between parallel first-terminating and coactive models, as both yield a positive MIC.

The cumulative distribution function on processing time for a single item, which is designated as the probability of cessation by time t, is equal to P(T ≤ t) = F(t). These functions or their complement, the survivor functions, where the latter is P(T > t) = 1 – F(t) were found to be considerably more powerful in uncovering the underlying combination of architecture and stopping rule (Townsend and Nozawa, 1995; Townsend and Wenger, 2004). For instance, a new statistical function of time, defined as a double difference of the survivor functions, now allowed for a differentiation of first-terminating parallel processing and coactivation as well as distinguishing between serial minimum time and serial exhaustive processing (Townsend and Nozawa, 1995; Townsend and Wenger, 2004).

This statistical function is known as the *survivor interaction contrast*, is defined as follows and can be evaluated for all RTs: *SIC*(*t*) = (*S*_*LL*_(*t*)−*S*_*HL*_(*t*))−(*S*_*LH*_(*t*)−*S*_*HH*_(*t*)) where *S(t)* denotes the survivor function [1−*F*(*t*)]. Observe that this is the same type of calculation as the MIC but now operates as an entire function of time. A positive SIC function is consistent with a parallel first-terminating, independent-channels model, whereas an SIC that first goes negative and then goes positive is predicted by a coactive model (see Townsend and Nozawa, 1995 for further detail). An ordering of S_*LL*_(t) > S_*HL*_(t) ≈S_*LH*_(t) > S_*HH*_(t) indicates that selective influence at the level of processing time distributions is not falsified.

#### Workload Capacity

A second aspect of SFT is the ability to elucidate the *workload capacity* [*C(t)*] of a system, which refers to the effects on efficiency as the workload is increased (e.g., processing two tones vs. processing one). Informally, the notion of *unlimited capacity* refers to the situation when the finishing time of a channel is identical to that of a standard parallel system. That is, the finishing times of the distinct subsystems are parallel and independent and their marginal finishing time distributions do not depend on how many others are engaged.^3^ The benchmark is to assess capacity (i.e., efficiency of processing speed) in comparison with standard parallel processing with specification of a particular stopping rule. The capacity coefficient, C(t) for an OR experimental design is defined in terms of the integrated hazard functions [H(t) = −ln(S(t))], with C(t) = H_AB_(t)/[H_A_(t) + H_B_(t)]. H_A_(t) is the integrated hazard function for item A and similarly for H_B_(t). H_AB_(t) is the corresponding integrated hazard function when both items are present. Thus, a capacity value of 1.0 is one in which the single-target RTs predict the dual-target RTs and performance is identical to that predicted by a standard parallel model.

Two alternative cases that differ from unlimited capacity are worth considering: capacity values below 1.0 (limited capacity) and those above 1.0 (super capacity). *Limited capacity* indicates that individual channels are processing at a rate slower than that of a standard parallel system. *Super capacity* indicates that individual channels are processing at a rate even faster than standard parallel processing. Observe that because C(t) is dynamic, the level of capacity could change across time.

The stopping rule affects overall processing times, so one must consider the architecture, the stopping rule, and the resource allocation of a system when evaluating RTs. A standard parallel system with an OR gate will predict decreases in mean RT as a function of the number of items undergoing processing (because all items are targets). However, we would consider this system unlimited, rather than super, capacity, as the predictions arise from a standard parallel model (i.e., unlimited capacity with independent channels).

In sum, this measuring instrument is that of the set of predictions by unlimited-capacity independent parallel processing (UCIP) which, as observed above is the class of standard parallel models. Again, *unlimited capacity* means here that performance is equivalent to a system where each parallel channel processes its input (item, etc.) just as fast when there are other surrounding channels working (i.e., with greater n) as when it is the only channel being forced to process information, and this is accomplished with independent channels. Naturally, because capacity and architecture are completely separate dimensions, though unlikely, even a serial system which tremendously increased its speed the more things it had to process could produce unlimited or even super capacity. We finally pause to emphasize that as with the tests for architecture (i.e., the survivor interaction contrast) our benchmark is a non-parametric and distribution-free class of models rather than a specific model based on a set of parameters.

### Applications of Systems Factorial Technology to the Auditory System

Lentz et al. (2016, 2017) applied SFT to the auditory system for the detection of one vs. two tones, with the 2016 study adopting monaural tone detection and the 2017 study adopting binaural tone detection. In both studies, Lentz et al. (2016, 2017) applied a standard double-factorial design, in which two tones of different frequencies (500 and 3,020 Hz) were presented singly or together at two stimulus levels (High = 80 dB SPL; Low = 38 dB SPL), with the experiment including all possible combinations of the two frequencies and levels, and included presentation intervals that contained silence. Although reaction times were measured to be faster for the High stimuli than the Low stimuli when the tones were presented singly, support for selective influence was not observed for the two-tone (dual-target) stimuli. That is, the reaction time distributions from the three of the four different dual-target stimuli (HH, HL, LH) were not differentiable for the majority of subjects. Although Lentz et al. (2016, 2017) did not find strict evidence of selective influence, selective influence was present at a mean level, and so, calculated SIC functions for their subjects. In 14 of 16 cases, SIC functions (and therefore also MIC values) were positive and suggested a parallel self-terminating architecture.

One explanation that would explain the lack of evidence for selective influence is related to a large body of work indicating that subjective loudness is a primary determinant of mean RT and that mean RT decreases with increasing loudness (c.f., Chocholle, 1940; Kohfeld et al., 1981; Humes and Ahlstrom, 1984; Schlittenlacher et al., 2014). We note that while brightness/light intensity is also a determinant for visual detection RTs in the visual system, these RTs are generally longer and change more rapidly with decreasing intensity than for the auditory modality (Kohfeld, 1971). Thus, the relationship between RT and loudness may contribute to the difficulty evincing selective influence, and perhaps making measurements in regions of this function where RTs change more drastically (e.g., at lower intensity levels; Kohfeld, 1971) might facilitate the ability to demonstrate selective influence. One advantage of RT studies over those that measure accuracy is that the mechanisms of perception can be evaluated at supra-threshold levels.

As pointed out earlier, outside of a very limited set of experiments, there is precious little in the literature that connects either para-threshold or even supra-threshold accuracy and scaling data with RT data or theory, particularly related to the detection of one vs. two sounds. Here, we attempt to measure system architecture at two overall sound levels so that we can (a) relate loudness to the RT measures of single-tone and double-tone stimuli across a range of sound levels and (b) put forth a following proof-of-concept in which we assume that processing times to detect sounds are driven by functions that are monotonically related to subjective loudness. The latter steps are in line with classical scaling theory of Fechner (1860) and Stevens (1957).

## Materials and Methods

### Stimuli

Stimuli were 350-ms 500 and 3,020-Hz pure tones having 25-ms cosine-squared onset and offset ramps. Stimuli were presented in two different scenarios: *SOFT*—tones presented at 35 (Low) and 50 (High) dB SPL and *LOUD*—tones presented at 65 (Low) and 80 (High) dB SPL. Note that the terms Low and High are relative within a scenario, referring to the relationship between the tone levels within either *LOUD* or *SOFT*.

### Procedures

Four observers, ranging in age from 19 to 22, participated in experimental sessions lasting 1–2 h. All subjects had hearing thresholds of 15 dB HL or better in both ears at standard audiometric frequencies. A single session consisted of 6–12 blocks of 128 trials. Each trial began with a visual warning of “listen” appearing on a computer monitor for 500 ms. Then, a random-duration, exponentially distributed, silent period with a mean of 300 ms followed removal of the warning, and the auditory stimulus was presented.

Table 1 illustrates the double-factorial OR design and the proportion of stimulus presentations used. One of two possible events occurred on each trial—a stimulus or a silent interval. Within each block of 128 trials, 25% contained the 500-Hz tone alone, 25% contained the 3,020-Hz tone alone, 25% contained dual-target stimuli: the 500 + 3,020-Hz tones, and 25% did not contain a stimulus. When single tones were presented, the High and Low stimulus levels were presented in equal proportions. For the dual-target stimuli, four stimuli were tested: HH, both tones at the High level; LL, both tones at the low level; HL the 500-Hz tone at the High level and the 3,020-Hz tone at the Low Level; and LH; the 500-Hz tone at the low level and the 3,020-Hz tone at the High level. Each of these events was a “Yes” trial. The remaining 25% of trials, no stimulus trials, were “No” trials.

**TABLE 1:** A list of the possible events in the double-factorial design, along with the frequency of each event.

**HH (6.25%)**
**HL (6.25%)**
**LH (6.25%)**
**LL (6.25%)**
**L500 (12.5%)**
**L3020 (12.5%)**
**H500 (12.5%)**
**H3020 (12.5%)**
No stimulus (25%)

75% of the trials are “Yes” trials, indicated in bold, whereas 25% of the trials are “No” trials, in which no stimulus was presented. The two-tone stimuli are indicated by HH, HL, LH, and LL, and single-tone stimuli are indicated by their level (H or L) and frequency in Hz.

Stimuli were presented using a randomized block design in which either *SOFT* or *LOUD* was selected randomly, and three blocks were run for that scenario. These scenarios were then alternated, with another three blocks tested before switching scenarios. A total of 12 blocks were collected for each scenario, with the first two blocks treated as practice. Thus, there were a total of 80 trials for each type of dual target (HH, LL, HL, and LH) and 160 trials for the single targets (H500, H3020, L500, and L3020).

Stimuli were presented to the observers at a 24,414 kHz sampling rate^4^ using a 24-bit Tucker Davis Technologies (TDT) hardware. Single-tone and two-tone stimuli were generated in Matlab and scaled appropriately prior to being loaded onto the TDT system. Stimuli were then played though a single channel of an RP2.1 real-time processor. A software clock within the TDT system (used to measure the RT) was triggered at the onset of the stimulus. Signals were then passed through a PA5 programmable attenuator and an HB6 headphone buffer. Sounds were presented to observers through the right earphone of a Sennheiser HD280 Pro headphone set. A button press using a box interfaced to the computer through the TDT serial port stopped the clock, to yield the RT.

Observers were instructed to respond as quickly to the signal tone as possible while attempting to provide correct responses. Using the “OR” design, observers were required to respond with the “yes” button if a tone was present. Otherwise, they were instructed to respond with the “no” button. The reaction time was measured from the onset of the tone stimulus. Percent correct was monitored in order to ensure that subjects achieved high levels of performance, and performance for all conditions was greater than 97.5%. Reaction times faster than 80 ms or slower than 800 ms have been removed from the data set.

## Results

### Reaction Times

Mean reaction time data from the single target trials are shown in Table 2 with the highest-level conditions in the leftmost columns. Generally, there is a clear increase in reaction time as intensity decreases. A repeated measures ANOVA conducted on each of the 500 Hz and the 3,020 Hz data revealed a statistically significant effect of level. For 500 Hz, the average decrease in RT from 35 to 80 dB was about 57 ms [*F*_(3, 9)_ = 33.8; *p* < 0.001], and at 3,020 Hz, the average decrease in RT from 35 to 80 dB was about 51 ms [*F*_(3, 9)_ = 10.0; *p* < 0.003]. At the individual levels, Kolmogorov-Smirnov tests supported a statistically significant difference in reaction time distributions at 500 Hz between 35 and 50 dB (k > 0.21; *p* < 0.001) and 65 and 80 dB for all subjects (k > 0.14; *p* < 0.03). For 3,020 Hz, three of the four subjects demonstrated significant faster RTs for 50 vs. 35 dB (k > 0.13; *p* < 0.05 except for S101 *k* = 0.13; *p* = 0.058) and 80 vs. 65 dB (k > 0.14; *p* < 0.04 except for S102 *k* = 0.08; *p* = 0.29).

**TABLE 2:** Mean RTs in ms for single targets in the two scenarios.

	*Condition*							
	*LOUD*				*SOFT*			
Subject	500H (80 dB)	3020H (80 dB)	500L (65 dB)	3020L (65 dB)	500H (50 dB)	3020H (50 dB)	500L (35 dB)	3020L (35 dB)
S100	190.9	187.6	209.1	195.0	223.0	206.8	232.5	223.9
S101	216.5	220.4	229.7	233.1	244.9	255.6	270.0	261.9
S102	185.4	218.2	206.3	225.8	231.9	254.3	269.5	312.5
S103	194.9	193.0	209.6	206.4	223.1	217.4	244.8	227.3
**Average**	**196.9**	**204.8**	**213.7**	**215.1**	**230.7**	**233.6**	**254.2**	**256.4**

Table 3 illustrates the mean reaction times in milliseconds for the dual-target stimuli. RTs generally follow the expected ordering (HH < HL = LH < LL), however, the mean RTs for HH, HL, and LH are very similar to each other. For example, for both scenarios, the average RTs in the HH condition are only 3–7 ms faster for HH compared to HL and LH, and individually, HH is not always faster than HL or LH. The largest average RT difference is between HH and LL in the *SOFT* scenario, and this difference is about 23 ms.

**TABLE 3:** Mean RTs in ms for the two-tone (dual-target) stimuli in the two scenarios.

	*Condition*							
	*LOUD*				*SOFT*			
Subject	HH	HL	LH	LL	HH	HL	LH	LL
S100	194.6	194.4	180.5	194.5	203.0	206.2	209.3	221.0
S101	205.6	215.2	216.0	216.2	234.8	232.8	241.2	256.3
S102	180.8	191.2	187.5	197.4	236.2	236.7	237.6	269.9
S103	185.5	193.0	188.5	197.8	204.9	214.3	215.9	223.8
**Average**	**191.6**	**198.5**	**193.1**	**201.5**	**219.7**	**222.5**	**226.0**	**242.7**

We conducted two repeated-measures ANOVAs to determine significant factors based on the group mean dual-target RTs. For the *SOFT* scenario, the ANOVA revealed a significant effect of condition [*F*_(3, 9)_ = 15.6; *p* < 0.001] whereas for the *LOUD* scenario, condition was not significant [*F*_(3, 9)_ = 3.7; *p* = 0.054]. To determine if selective influence at the individual level held for the two scenarios, we conducted four required one-tailed Kolmogorov-Smirnoff tests: HH < HL, HH < LH, HL < LL, LH < LL. For the *SOFT* scenario, no subject met the criteria for selective influence, and S103 met the criteria for selective influence in the *LOUD* scenario. Thus, even though RTs differed more in the *SOFT* scenarios than the *LOUD* ones, evidence for selective influence at the individual distributional level was not more prevalent in *SOFT* vs. *LOUD*.

### Individual Data: Dual Targets

**FIGURE 2:**
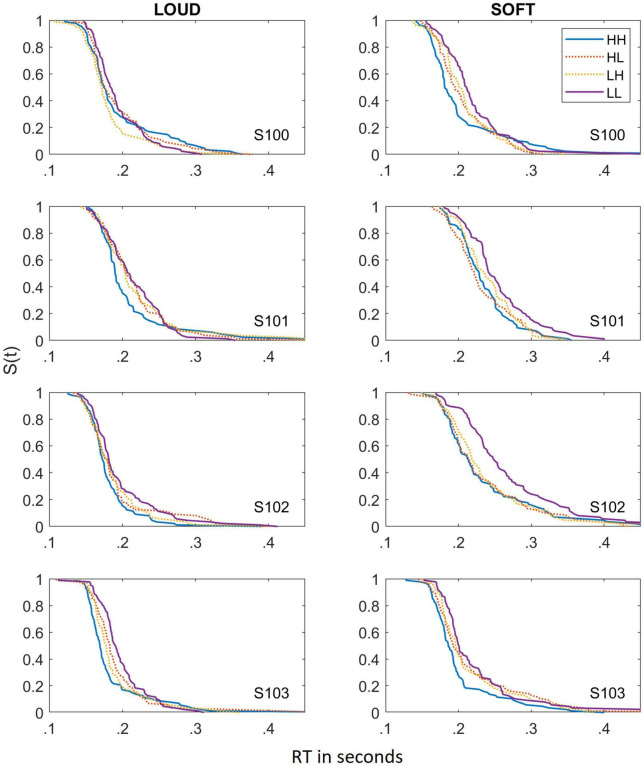
Survivor functions obtained for the dual-target stimuli for the LOUD scenario **(left panels)** and the SOFT scenario **(right panels)**. Data from the different dual-target conditions (HH, HL, LH, and LL) are depicted by lines of different colors and solid lines indicate HH and LL whereas dotted lines reference HL and LH.

To better illustrate the reasons behind the failure of selective influence for the dual targets, the survivor functions based on the estimated cumulative distribution functions for all four subjects in the two different scenarios are shown in Figure 2. Although a primary factor is that the means of the different conditions are very similar to each other, selective influence also fails because the survivor functions cross at some point. For example, S101 in the *SOFT* scenario illustrates faster RTs at the slow RTs for the HL condition, but once the RTs are slower (e.g., about 230 ms), RTs are faster in the HH compared to the HL condition. In many cases, the survivor functions for HH, HL, and LH all appear to be essentially the same (e.g., S102 in the *SOFT* scenario). The means in which selective influence fails appears to be very similar for the *LOUD* and *SOFT* scenarios. Due to the broad failure of selective influence in both scenarios, we do not report SIC functions for these data.

### Capacity

**FIGURE 3:**
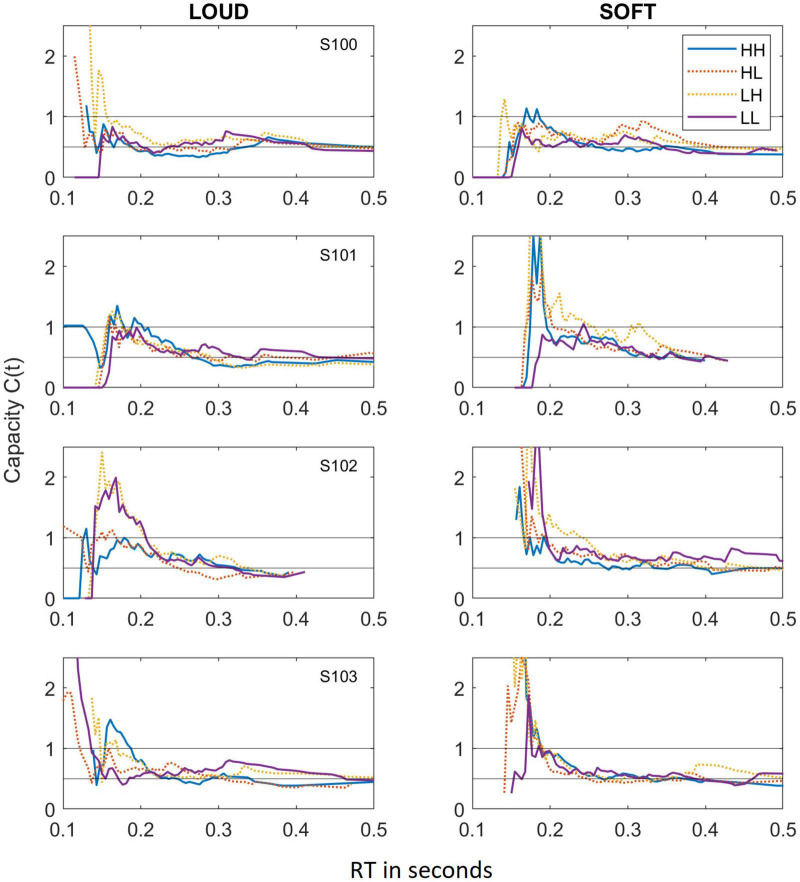
Capacity functions obtained for the LOUD scenario **(left panels)** and the SOFT scenario **(right panels)**. As in Figure 2, data from the different dual-target conditions (HH, HL, LH, and LL) are depicted by lines of different colors and solid lines indicate HH and LL whereas dotted lines reference HL and LH.

Figure 3 shows capacity functions for the two different scenarios tested: *LOUD* and *SOFT*.

As we have observed in other experiments on auditory detection (e.g., Lentz et al., 2016, 2017), the capacity functions appear to be dynamic, with high capacity values occurring for short RTs and much lower capacity illustrated at the longer RTs. The majority of data points fall within C(t) values of 1.0 (unlimited capacity) and 0.5 (fixed capacity), and while some capacity values exceed 1.0, these regions are typically based on only a few RTs and are therefore not necessarily indicative of super capacity. In fact, the statistical package offered by Houpt et al. (2013), finds that the majority of these capacity functions are statistically different from UCIP and are limited capacity (Houpt and Townsend, 2012). In four cases, capacity is not statistically different from UCIP, and those cases are illustrated in Table 4, which shows the mean capacity values for each subject and condition, in bold and italicized text.

**TABLE 4:** Mean capacity values for the different conditions tested.

	*Condition*							
	*LOUD*				*SOFT*			
Subject	HH	HL	LH	LL	HH	HL	LH	LL
S100	0.51	0.57	0.71	0.51	0.45	0.57	0.55	0.44
S101	0.62	0.51	0.48	0.52	0.74	0.78	***1.18***	0.61
S102	0.55	0.71	***0.86***	***0.79***	0.59	0.77	***0.87***	0.86
S103	0.56	0.72	0.73	0.98	0.82	0.75	0.86	0.69
**Average**	**0.56**	**0.62**	**0.70**	**0.70**	**0.64**	**0.71**	**0.88**	**0.65**

Capacity values that are bold and italicized are not statistically different from the UCIP model.

## Relationship Between RT and Loudness

As in Lentz et al. (2016, 2017) we again have observed a failure of selective influence and the presence of limited capacity for the detection of one vs. two tones, and we did not evince more evidence of selective influence when RTs were measured at lower overall levels (i.e., in the *SOFT* scenario). We now put forth a hypothesis that these observations are consistent with a perceptual mechanism in which processing times to detect sounds are driven by psychophysical functions that are monotonically related to subjective loudness and suffice to squash the distributions of the faster times (i.e., HH, HL, and LH) to the extent that we witness a failure of selective influence. We discuss two main pieces of evidence to support this hypothesis.

**FIGURE 4:**
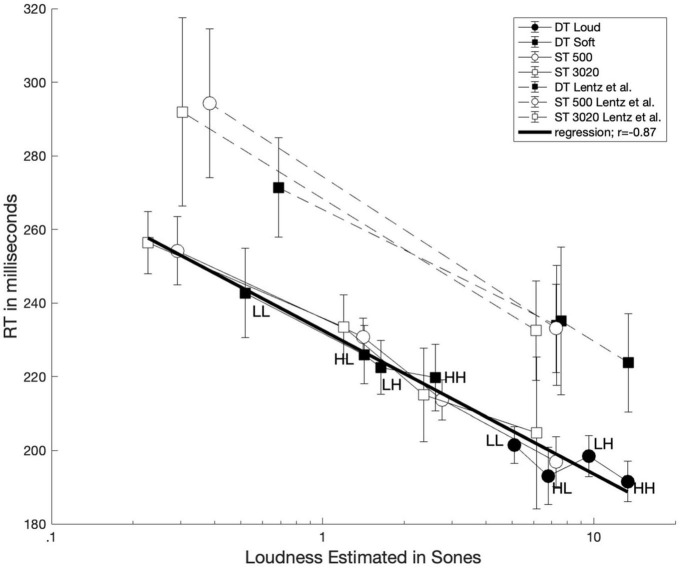
Average reaction time plotted as function of loudness estimated from Moore and Glasberg’s loudness model. Data from single-target and dual-target conditions are illustrated with unfilled and filled symbols, respectively. The solid dark line represents a log-linear regression line using the current data set.

First, we evaluated whether the loudness of these sounds is related to mean RT. One might already expect that this has been well-established, but we are not aware of any studies relating reaction time to loudness for two-tone stimuli. Although we did not measure loudness directly, we used the validated model of Moore et al. (1997) and International Organization for Standardization (2017), which has enjoyed considerable success in calculating the loudness of steady sounds. We purport that it is also sensible to use a loudness model to estimate loudness, due to difficulty and time-consuming nature of implementing scaling techniques. The loudness (in sones) of the stimuli calculated from the model as implemented by Moore et al. (2016; using the “middle-ear only” correction) are plotted vs. the measured reaction times in Figure 4. Data from the dual-target conditions are represented with filled symbols, whereas data from the single-target conditions are represented with unfilled symbols. For comparison, results from Lentz et al. (2016) are also shown with dashed lines. A log-linear regression was conducted between estimated loudness and RTs for the current data set (*r* = −0.87). The best-fitting line is plotted on Figure 4 as a dark solid line.

Figure 4 illustrates a strong relationship between the modeled loudness of the stimuli and the associated reaction times. We also note that the loudness of the dual-target stimuli, particularly HL, LH, and HH, are rather similar. This feature could explain why the RTs are also similar for these conditions and thus may lead to difficulty observing selective influence. The strong correlation between loudness and RT lends credence to the hypothesis that RT is indeed driven by loudness for both one-tone and two-tone stimuli.

## Merging Loudness Growth With Systems Factorial Technology Predictions

Second, we posit that loudness-driven RTs would also be consistent with limited capacity given the findings of Lentz et al. (2016). There are several major and quite distinct ways in which a set of parallel channels can evince limited capacity. The first two have been studied and employed broadly (e.g., Townsend and Ashby, 1978, 1983; Townsend and Wenger, 2004; Eidels et al., 2011; Algom et al., 2015). A third is offered here for the first time and falls into the class of integrated subadditive parallel systems and are a form of a coactive system. These three are discussed here, with greater attention to be focused on the latter explanation.

(1). *Limited Resource Parallel Systems*: In this model, there is simply a limited pool of resources which must be shared among an active set of channels. Such systems perform at levels varying from extremely limited [e.g., *C(t)* ≈0] up to mildly limited [*C(t)* ≈1], A special case of import is that of *fixed capacity*, *C(t)* = *C*_*f*_ but with stochastically independent channels. With n channels, with an equal spread of capacity, *C*_*f*_ = 1/n. Such a system produces the same level of performance as a standard serial system in OR designs (i.e., very poor!).
(2). *Mutually Inhibitory Parallel Systems*: The channels arranged in parallel inhibit one another. Such systems typically are not stochastically independent but rather possess negative dependencies, as illustrated in Figure 1.
(3). *Integrated Subadditive Parallel Systems:* As introduced earlier, an integrated type of system combines activation from the two (or more) channels into a single late conduit. We must consider two very distinct cases. One transpires when each channel’s activation is a stochastic (i.e., probabilistic in time) random variable and the final conduit sums the two independent random variables. If the activations are additive and the integrated hazard function is based on that summed random variable, we have the now-classic case of a coactive system (Miller, 1978; Schwarz, 1989; Diederich and Colonius, 1991; Townsend and Nozawa, 1995; Houpt and Townsend, 2011). These types of systems evidence extreme super capacity, i.e., *C*(*t*)≫1.However, another possibility arises when the input activation is a constant related to (or being!) the psychophysical function and the distribution is found by multiplying that constant with the inherent hazard function. If the combined signal (represented by the psychophysical function) is subadditive (e.g., if the exponent in Steven’s Power Law is less than 1: *S* = *KI^p^*; Stevens, 1957, *p* < 1) and the integrated hazard function is proportional (at least) to the combination, limited capacity will result. The opposite occurs if *p* > 1.

Given the novelty and relative unfamiliarity of the power law in theory and methodology of response times, we take some space here to outline a *proof of concept.* We thereby demonstrate, assuming a classic subadditive psychophysical function, that for higher values of intensity the survivor functions draw closer together and thereby provide a potential explanation for the statistical failure of selective influence in an SFT experiment. We then turn our attention to the notion of capacity, which also illustrates a limited nature.

First, let ψ(I, K) stand for the psychophysical increasing function of stimulus intensity, where I stands for encoded stimulus intensity and K is a set of parameters (perhaps just one) needed to predict human performance. So, *d*ψ/*dI* > 0 indicating that the loudness sensation increases with intensity. For now, we assume that ψ_*f*1_ = ψ_*f*2_ where f_1_ and f_2_ refer to the two different frequencies constituting the two signals. Then, suppose S(t, ψ, K) stands for a survivor function that is a decreasing (as should be the case) function of ψ, *dS*/*d*ψ < 0. This statement states that the likelihood that the stimulus has not been detected by time t decreases with intensity.

Note that we simply consider that I is the germane factor for SFT purposes. Now assume that subjective loudness is some conjoint function of I_f1_ and I_f2_, meaning that the two signals are merged into one. Letting I_*f*1_, I_*f*2_ represent the encoded intensities from the two frequencies, we write this as ψ (I_*f*1,_ I_*f*2_) for now, increasing in each argument. Then, we move to formulate our prime axioms. Thus, we postulate that for all t ≥ 0, $\frac{d\,S}{d\,\psi }<0\,\left(a\,s\,a\,b\,o\,v\,e\right)$, and *d*^2^*S*/*d*ψ^2^ > 0. The latter condition is needed to reflect our belief that as ψ grows, the survivor function slows down its descent, which means the survivor curves get closer together as intensity is increased. More formally, we have:

*PROPOSITION I:* If the above postulates are in force, the survivor functions of I become ever closer as I grows larger.

*PROOF:* The inference is immediate because *dS*/*d*ψ < 0 implies the proper decreasing behavior that the process has not finished by time t, as I increases and *d*^2^*S*/*d*ψ^2^ ensures that the rate of decrease is ever slowing down as I increases. This behavior occurs for all times, t.

Next, we postulate that incoming sensory stimulation is summed so that the psychophysical function for two tones follows the same form as for a single tone. We further assume that the effect of this summed quantity follows Stevens’ Power Law, that is, ψ (I_f1_, I_f2_, K) = K (I_f1_ + I_f2_)*^p^* where K > 0 and 0 < p < 1 for loudness (Stevens, 1957). Since *p* < 1, the overall effect of ψ is to act as a squashing function.

To evaluate SIC functions, we now examine the double difference in terms of I_*f*1_, I_*f*2_, which we can interpret as our SFT factors. Recall for our earlier presentation that we failed (Lentz et al., 2016, 2017 and here) to find the required selective influence S_HH_(t) < S_HL_(t) ≈ S_LH_(t) < S_LL_(t), in that our data indicated S_HH_(t) ≈ S_HL_(t) ≈ S_LH_(t). The implication here is that if we were able to evince selective influence, the associated SIC function would also be positive for all t. But, our hypothesis is that the latter three distributions are so close together for the usual stimulus sets, that our sample sizes are too small to detect a significant difference.

To show this mathematically, first we need to connect ψ with a stochastic representation by rendering assumptions about how ψ affects, say, the time to accrue evidence up to a criterion. Recall that *any* survivor function on time (equivalently, on any positive valued random variable) can be expressed as Exp[-H(t)] where H(t) is the integrated hazard function.

Additional complexity at this point does little good (that is, would only add more untestable conditions), so we make the very simplifying assumption that the integrated hazard function associated with loudness is proportional to ψ (I_f1_, I_f2_, K) x t^d^.^5^

This trick delivers us the pleasant feature that our survivor functions are Weibull with rate proportional to ψ: *S*_*f*1*f*2_(*t*,*p*,*a*) = *e*^−*a*ψ(*I*_*f*1_,*I*_*f*2_,*K*)*t^d^*^ = *e*^−*aK*(*I*_*f*1_+*I*_*f*2_)*^p^t^d^*^, where K > 0, a > 0, and 0 < p < 1, d > 0, where f_1_ and f_2_ refer to the two channels in the processing architecture and d is the traditional shape parameter^6^.

*PROPOSITION II* Set ψ(*I*_*f*1_,*I*_*f*2_,*K*) = *K*(*I*_*f*1_+*I*_*f*2_)*^p^*, K > 0, a > 0, and 0 < p < 1. This psychophysical function of input from the two channels satisfies *S*_*LL*_(t, p, a) -*S*_*LH*_(t,p,a) – [*S*_*HL*_(t,p,a) - *S*_*HH*_(t,p,a)] > 0 for all t ≥ 0, and therefore under this model, SIC functions should be positive.

Following the logic of Townsend and Nozawa (1995), when taken at the infinitesimal level, this statement is equivalent to, implementing the chain rule,


$$\frac{\partial^{2}S_{f\,1\,f\,2}\,\left(t,I_{f\,1},I_{f\,2},p,a\right)}{\partial I_{f\,1}\,\partial I_{f\,2}}=\left(d^{2}\,S/d\,\psi^{2}\right)\,\left(\partial \psi /\partial I_{f\,1}\right)\,\left(\partial \psi /\partial I_{f\,2}\right)+\left(\frac{d\,S}{d\,\psi }\right)\,\left(\frac{\partial^{2}\psi }{\partial I_{f\,1}\,\partial I_{f\,2}}\right)$$

PROOF: Now, d^2^*S*/dψ^2^>0 by virtue of *S*_*f*1*f*2_(*t*,*p*,*a*) = *e*^−*a*ψ(*I*_*f*1_,*I*_*f*2_,*K*)*t^d^*^, ∂ψ/∂ I_*f1*_>0 and ∂ψ/∂I_*f*2_ > 0 by assumption, (d*S*/dψ) <0 also by *S*_*f*1*f*2_(*t*,*p*,*a*) = *e*^−*a*ψ(*I*_*f*1_,*I*_*f*2_,*K*)*t^d^*^, and finally it is straightforward to see that $\frac{\partial^{2}\psi }{\partial I_{f\,1}\,\partial I_{f\,2}}>$ 0 by virtue of 0 < p < 1. This sum of products is always positive, which implies that *S*_*LL*_(t, p, a) -*S*_*LH*_(t,p,a) – [*S*_*HL*_(t,p,a) - *S*_*HH*_(t,p,a)] > 0 for all t ≥ 0 as we wished. Thus, the proposition is proven.

The conclusion is that the three functions S_*LH*_ (t) ≈ S_*HL*_(t) ≈ S_*HH*_(t) may become increasingly indistinguishable as a function of I, in the presence of statistical error. We can usually, again for our purposes, explicitly put down an explicit form for ψ and then bypass the chain rule.

**FIGURE 5:**
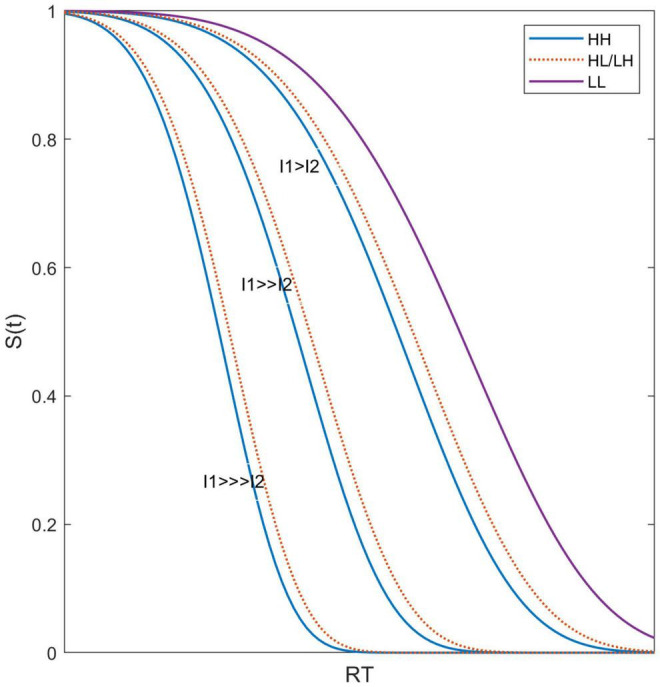
Predicted survivor functions illustrating that the effects of the difference in stimulus intensity between the two targets leads to survivor functions for HH, HL, and LH being very similar to each other.

Example survivor functions based on this simple model are illustrated in Figure 5. Letting *p* = 0.3, the usual power as typically measured for sound intensity (*S* = *KI*^.3^), Figure 5 shows the survivor functions for three relationships of I (For simplicity, I_*i*_ = I_*i*_): I_1_>>>I_2_ (50 dB difference), I_1_>>I_2_ (30 dB difference), and I_1_ > I_2_ (10 dB difference), and I_2_ is fixed. For illustration purposes, K, a, and d are set to arbitrary values to best illustrate these effects (1, 0.01, 5, respectively), noting that for our goals, only the basic form of what is happening is pertinent. We note that under this model, HL and LH survivor functions are identical. Notably, Figure 5 illustrates that HH, HL, and LH survivor functions are much more similar to each other than to the LL survivor functions.

Besides architecture and decisional stopping rule and as pointed out in our Introduction, another key attribute of processing systems is the degree of efficiency as the workload is varied or what goes by the term “workload capacity” in SFT. We now show that the Stevens power law as interpreted above predicts super, unlimited or limited capacity depending on whether *p* > 1 (super), *p* = 1 (unlimited), or *p* < 1 (limited). In our elementary form, the integrated hazard function (an exponential distribution with “rate” = psychophysical function) is just the product of t and Stevens power function: t ⋅ (I_*f*1_+ I_*f*2_)*^p^*.

*PROPOSITION III:* Workload capacity attending our Stevens Power Law Model is super, unlimited, or limited according to the values of *p* < 1, *p* = 1, or *p* > 1, respectively.

*PROOF:* (I_*f*1_ + I_*f*2_)*^p^* is overadditive, additive, or subadditive relative to (I_*f*1_*^p^* + I_*f*2_*^p^*) according to the magnitude of *p* > 0. Thus, C(t) = (I_*f*1_ + I_*f*2_)*^p^* /(I_*f*1_*^p^* + I_*f*2_*^p^*) and the numerator is greater than, equal to or less than the denominator depending on whether *p* > 1 (super), *p* = 1 (unlimited), or *p* < 1 (limited).

**FIGURE 6:**
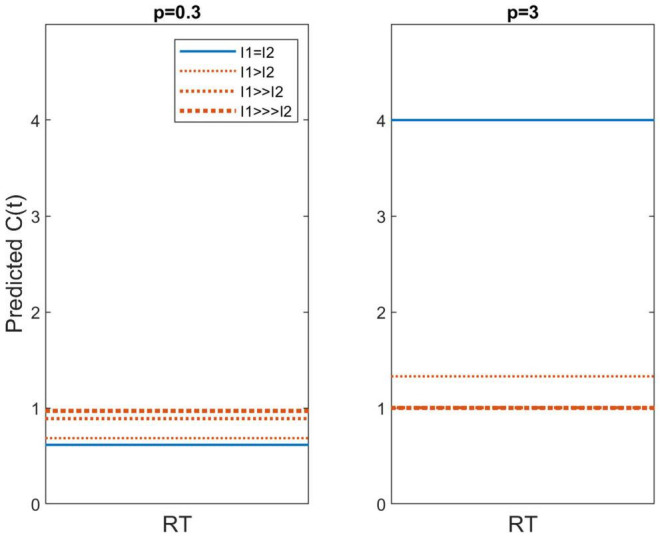
Predicted capacity functions for *p* = 0.3 and *p* = 3. Conditions in which I1 = I2 (i.e., HH and LL) are shown as the blue solid line whereas predictions for I1≠I2 (i.e., HL and LH) are shown with orange dotted lines.

Figure 6 exhibits C(t) for *p* = 0.3 in the left panel and *p* = 3 in the right panel. The latter yields super capacity values for the I_1_ = I_2_ conditions. So far, capacities this high are relatively rare. For instance, Houpt et al. (2014) found that word and pronounceable non-words possessed much higher capacity than upside down English letter sequences and Katakana strings but even the former C’s were pretty well bounded below C(t) = 4, although the latter were very poor, asymptoting around C(t) ≈.1. These predictions illustrate that this model also predicts different capacity values for LH and HL conditions. When p > 1, the power function leads to a higher capacity value and the opposite occurs for *p* < 1. Should *p* = 1, we have the specific case of unlimited capacity, with all C(t) values equal to 1.

At the established value of *p* = 0.3 we find C(t) ≈0.6, which is very similar to the capacities observed in this study. However, the current data do not provide robust evidence that C_*HL*_ or C_*LH*_ > C_*LL*_ or C_*HH*_, although the levels used in these experiments are only 15 dB apart. On the other hand, Lentz et al. (2017) used stimulus levels over 40 dB apart and they also did not report clear evidence of higher capacity values for HL and LH compared to HH and LL.

More generally, an arbitrary integrated hazard function, say, H[t, ψ(I_*f*1_, I_*f*2_)] can be assessed as to whether it is an overadditive (therefore super capacity), additive (therefore unlimited capacity), or subadditive (therefore limited capacity) relative to H[t, ψ(I_*f*1_)] + H[t, ψ(I_*f*2_)].

## Conclusion

It is well established that mean RT is a decreasing function of loudness. However, it is another question as to whether an ordering of distributions on RT as a function of intensity holds at a stronger level (see, e.g., Townsend, 1990). Moreover, such issues that arise when one considers the sum of two or more tones, possibly of different frequencies, also have remained unanswered, although some recent studies suggest that sums of tones may not be highly effective in shortening RTs (e.g., Schröter et al., 2007, 2009; Fiedler et al., 2011; Lentz et al., 2016, 2017).

We found ourselves compelled to open (or reopen) these issues when our earlier attempts to employ distributional orderings to affirm selective influence in a study on RT as a function of the intensities of two distinct frequencies was unsuccessful. Successful selective influence would have ordered the respective survivor functions as HH < HL ≈ LH < LL but in fact, HH ≈ HL ≈ LH < LL in terms of statistical significance. The experimental part of the present effort replicated those findings of a failure to satisfy selective influence, and such findings were evident when measured at both relatively low (soft) and relatively high (loud) stimulus levels. We again observed limited capacity in comparing the detection of one and two tones (e.g., Schröter et al., 2007; Lentz et al., 2016, 2017). Our analyses indicated that the loudness of stimuli (whether they be one or two tones) did produce monotonically related mean reaction times.

We presented a *proof-of-concept* model which, based on an assumption that processing times to detect sounds are driven by psychophysical functions of tonal loudness as functions of intensity, predicted both experimental observations. This model is consistent with the findings of Schröter et al. (2007, 2009) who have argued that when two tones are fused together into a single percept, a redundant signals effect is not observed. At this point in time, we believe that the above analytic efforts and illustrative computations are sufficient to render the integrated subadditive psychophysical function (in this case, employing a Stevens power law with a fractional exponent) a suitable contender for our failure of dominance as well as our finding of limited capacity. This is the first demonstration that an integrated parallel subadditive model may provide an appropriate description of auditory detection architecture.

One way of thinking about our experimental design is as a so called redundant signals paradigm. Such experiments recede into the past (see, e.g., Egeth, 1966; Bernstein, 1970). However, when Miller (1978) initiated a novel upper bound to assess how much RTs were speeded up (or not) in a bimodality study, many researchers, especially in the field of multimodal perception, began to apply the bound (i.e., an inequality on RT), and such work has been conducted over several decades. It has often been found, as in Miller’s first studies, that capacity with combined vision and sound is at a sufficiently high capacity that the Miller bound is violated (e.g., Fox and Houpt, 2014). None of our observers even reached capacity significantly above the unlimited prediction and all asymptoted close to the lower bound known as *fixed capacity* (Townsend and Nozawa, 1995). It was therefore impossible that the upper (Miller) bound could have been violated.

In addition to the potential causes of limited capacity we outlined earlier, Miller put forth a model that could produce in between levels of performance (Miller, 1988), the *grains model* (Miller and Ulrich, 2003). If most of the grains are shared by two signals, the output would be like hearing a single tone (and therefore close to fixed capacity in our terms) but if they are not shared, performance would approach that of a (super capacity) coactive model (see also Schröter et al., 2009 who applied this model to similar stimuli). It may be that this concept could be expanded into a model for the kind of result we have found with our survivor functions.

In a sense, the simplest explanation of all would be that we still have a parallel race going on but that selective influence is simply weaker as sounds intensify. Recall that independent race models (whatever their capacity) will predict that the difference of the respective survivor functions will decrease from S_*LL*_(t) to S_*LH*_(t) and S_*HL*_(t) and from the latter to S_*HH*_(t). If there is too much “noise” at the bottom (as in Figure 2), selective influence will not be detectible there. What would *not work* would be to have such a race and try to coax the system into the phenomena on which we deliberate by letting each channel’s subjective intensity be governed by a Stevens’ scale: Such a system will, perforce, exhibit unlimited capacity!

Another consideration is that of the quality of the sound itself, as also noted by Schröter et al. (2007, 2009), and whether the two sounds merge into a single percept. Schröter et al. (2007, 2009) argued that two tones merged into a single percept (which yielded very slow dual-target RTs) whereas a tone and a noise did not (and therefore yielded much faster dual-target RTs). Yet, we suppose it is possible, in theory, that one could still dissect the signal into separate sounds and yet have the loudness of the combined stimulus be the Stevens’ function we have posited. Such issues are fascinating to us and may require significant effort to untangle.

We are not in favor, at this juncture in time, to attempt more detailed parameter estimation and utilization and model fitting. We rather advocate that researchers endeavor to construct appropriate experimental designs that can test among the above options. In addition it would be valuable to explore new paradigms in the auditory domain that permit enhanced selective influence and therefore, to truly assess architecture and stopping rules. It has turned out in visual cognition that other aspects of stimuli than simply intensity can drive effective selective influence. For instance, degrees of similarity among even fairly complex or configural features (e.g., distance between the eyes in faces; Fifić and Townsend, 2010) can serve as valid selective influence dimensions. We posit that it is likely that something like this can be applied to auditory perception.

Finally, we suggest that psychophysics of sound as a discipline might be reinvigorated by bringing to bear the burgeoning sets of tools available in the field of mathematical modeling and in particular, those instruments connected with the analysis of RTs.

## Data Availability Statement

The raw data supporting the conclusions of this article will be made available by the authors, without undue reservation.

## Ethics Statement

The studies involving human participants were reviewed and approved by the Indiana University Institutional Review Board. The patients/participants provided their written informed consent to participate in this study.

## Author Contributions

JL and JT collaborated on the manuscript. JL contributed to the design and implementation of the experiments, data collection and analysis, and primary writing of the manuscript. JT contributed to the design of the experiments, the analytical modeling, and also the writing of the manuscript. Both authors contributed to the article and approved the submitted version.

## Conflict of Interest

The authors declare that the research was conducted in the absence of any commercial or financial relationships that could be construed as a potential conflict of interest.

## Publisher’s Note

All claims expressed in this article are solely those of the authors and do not necessarily represent those of their affiliated organizations, or those of the publisher, the editors and the reviewers. Any product that may be evaluated in this article, or claim that may be made by its manufacturer, is not guaranteed or endorsed by the publisher.

## Footnotes

1 These kinds of phenomena first occurred in fields focusing on high accuracy RTs when two or more signals (i.e., so called redundant signals conditions) were presented vs. when a single (i.e. a target) was presented (e.g., Miller, 1978; Diederich and Colonius, 1991; Townsend and Nozawa, 1995). In that body of research, this type of model is known as *coactive*. These topics will be explained in greater detail in what follows.

**footnote:** ^2^By now, SFT has been applied to almost all major disciplines of psychology and cognitive science. Introductions are now widely available, for instance, in Algom et al. (2015); Harding et al. (2016), and Townsend et al. (2018). Up to date applications and surveys can be found in Little et al. (2017) and Houpt et al. (2019).

3 Observe that theoretically, probability summation could also result from a serial process if accuracy alone is recorded. Thus, observers would garner their two samples, one from each signal possibility and base their response on the maximum observation.

4 The unusual sampling rate is one of the preset sampling rates within the TDT hardware.

5 A quite pleasant feature of RT modeling is that any distribution represented by a survivor function on a positive random variable (here, time) can be written uniquely as S(t) = *e*^−*H*(*t*)^. H(t) is called the integrated hazard function because it equals $\int_{0}^{t}h\,\left(t^{\prime }\right)\,dt^{\prime }$ where h(t’) is the actual hazard function itself and represents the instantaneous likelihood that the process completes, given that it hasn’t finished yet. This then propels H(t) = – ln[S(t)].

6 We are definitely *not* saying that *p* < 1 for all modalities, just for sound intensity here.
